# *cis*-(Nitrato-κ^2^ *O*,*O*′)(2,5,5,7,9,12,12,14-octa­methyl-1,4,8,11-tetra­aza­cyclo­tetra­decane-κ^4^ *N*,*N*′,*N*′′,*N*′′′)cadmium nitrate hemihydrate

**DOI:** 10.1107/S160053681201238X

**Published:** 2012-03-28

**Authors:** Tapashi G. Roy, Saroj K. S. Hazari, Babul Chandra Nath, Seik Weng Ng, Edward R. T. Tiekink

**Affiliations:** a Department of Chemistry, University of Chittagong, Chittagong 4331, Bangladesh; b Department of Chemistry, University of Malaya, 50603 Kuala Lumpur, Malaysia; c Chemistry Department, Faculty of Science, King Abdulaziz University, PO Box 80203 Jeddah, Saudi Arabia

## Abstract

The Cd^II^ atom in the title complex, [Cd(NO_3_)(C_18_H_40_N_4_)]NO_3_·0.5H_2_O, is coordinated within a *cis*-N_4_O_2_ donor set provided by the tetra­dentate macrocyclic ligand and two O atoms of a nitrate anion; the coordination geometry is distorted octa­hedral. The lattice water mol­ecule is located on a twofold rotation axis. N—H⋯O hydrogen bonds and weak C—H⋯O inter­actions link the complex cations into a supra­molecular layer in the *bc* plane. Layers are connected by O—H⋯O hydrogen bonds between the lattice water mol­ecule and the non-coordinating nitrate anion, as well as by weak C—H⋯O contacts.

## Related literature

For background to macrocyclic complexes, see: Hazari *et al.* (2008 ▶). For the crystal structure of the anhydrous form of the title complex, see: Hazari *et al.* (2010 ▶). For the synthesis of the macrocyclic ligand, see: Bembi *et al.* (1989 ▶).
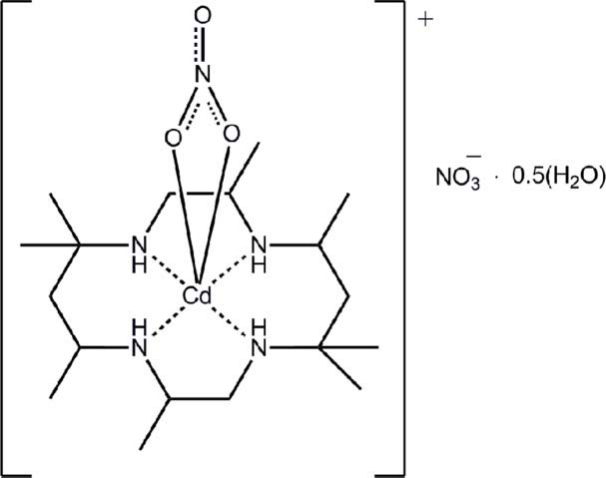


## Experimental

### 

#### Crystal data

[Cd(NO_3_)(C_18_H_40_N_4_)]NO_3_·0.5H_2_O
*M* *_r_* = 557.98
Monoclinic, 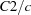

*a* = 18.4312 (4) Å
*b* = 11.3595 (2) Å
*c* = 25.1662 (6) Å
β = 111.782 (3)°
*V* = 4892.8 (2) Å^3^
*Z* = 8
Cu *K*α radiation
μ = 7.55 mm^−1^
*T* = 100 K
0.15 × 0.15 × 0.15 mm

#### Data collection

Agilent SuperNova Dual diffractometer with an Atlas detector
Absorption correction: multi-scan (*CrysAlis PRO*; Agilent, 2011 ▶) *T*_min_ = 0.738, *T*_max_ = 1.000
8065 measured reflections
4713 independent reflections
4467 reflections with *I* > 2σ(*I*)
*R*_int_ = 0.019

#### Refinement

*R*[*F* ^2^ > 2σ(*F* ^2^)] = 0.029
*wR*(*F* ^2^) = 0.078
*S* = 1.06
4713 reflections
289 parameters
1 restraint
H atoms treated by a mixture of independent and constrained refinement
Δρ_max_ = 0.81 e Å^−3^
Δρ_min_ = −0.63 e Å^−3^

### 

Data collection: *CrysAlis PRO* (Agilent, 2011 ▶); cell refinement: *CrysAlis PRO*; data reduction: *CrysAlis PRO*; program(s) used to solve structure: *SHELXS97* (Sheldrick, 2008 ▶); program(s) used to refine structure: *SHELXL97* (Sheldrick, 2008 ▶); molecular graphics: *ORTEP-3* (Farrugia, 1997 ▶) and *DIAMOND* (Brandenburg, 2006 ▶); software used to prepare material for publication: *publCIF* (Westrip, 2010 ▶).

## Supplementary Material

Crystal structure: contains datablock(s) global, I. DOI: 10.1107/S160053681201238X/xu5483sup1.cif

Structure factors: contains datablock(s) I. DOI: 10.1107/S160053681201238X/xu5483Isup2.hkl

Additional supplementary materials:  crystallographic information; 3D view; checkCIF report

## Figures and Tables

**Table 1:** Selected bond lengths (Å)

Cd—O1	2.4562 (19)
Cd—O2	2.404 (2)
Cd—N1	2.306 (2)
Cd—N2	2.307 (2)
Cd—N3	2.303 (2)
Cd—N4	2.312 (2)

**Table 2:** Hydrogen-bond geometry (Å, °)

*D*—H⋯*A*	*D*—H	H⋯*A*	*D*⋯*A*	*D*—H⋯*A*
O1*w*—H1*w*⋯O5	0.85 (1)	2.04 (2)	2.836 (3)	156 (5)
N1—H1*n*⋯O1^i^	0.88	2.42	3.242 (3)	155
N2—H2*n*⋯O4	0.88	2.30	3.133 (4)	157
N4—H4*n*⋯O5	0.88	2.11	2.991 (3)	175
C5—H5*B*⋯O6^ii^	0.98	2.58	3.539 (5)	168
C11—H11⋯O4^iii^	1.00	2.44	3.358 (3)	152
C9—H9*B*⋯O1*w*^iv^	0.98	2.51	3.451 (4)	162

Symmetry codes: (i) 
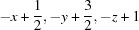
; (ii) 
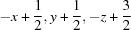
; (iii) 
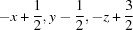
; (iv) 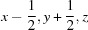.

**Fig. 1.**
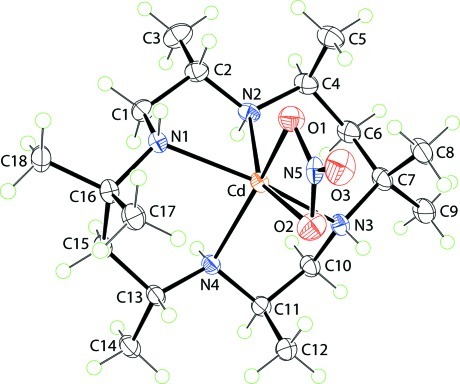
The molecular structure of the cation in (I) showing the atom-labelling scheme and displacement ellipsoids at the 50% probability level.

**Fig. 2.**
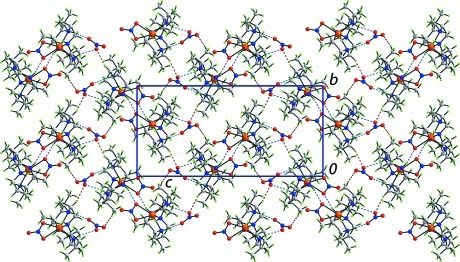
A view of the supramolecular layer in the bc plane in (I). The N—H···O hydrogen bonds and C—H···O interactions are shown as blue and brown dashed lines, respectively.

**Fig. 3.**
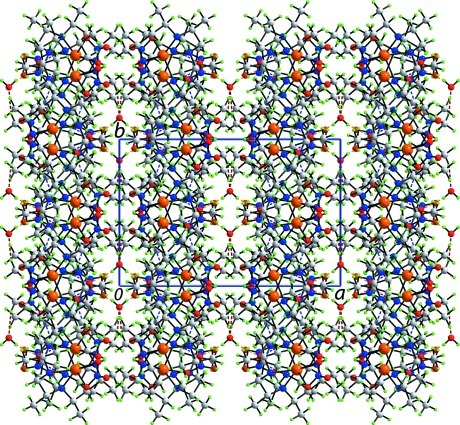
A view of the unit-cell contents in projection down the c axis in (I). The O—H···O, N—H···O hydrogen bonds and C—H···O interactions are shown as orange, blue and brown dashed lines, respectively.

## supplementary crystallographic information

### Comment

In continuation of on-going studies of the synthesis, characterization and biological activities of substituted tetraazamacrocyclic ligands and their metal complexes (Hazari *et al.*, 2008), attention was directed to cadmium complexes (Hazari *et al.*, 2010). In that study, the title complex was investigated in its anhydrous form. Recently, it was isolated as a hemihydrate (I). Herein, we describe the crystal structure of (I).

In cation in (I), Fig. 1, the Cd^II^ atom exists within a *cis*-N_4_O_2_ donor set defined by the four nitrogen atoms of the macrocyclic ligand and two nitrate-O atoms, Table 1. The coordination geometry is based on an octahedron, but with significant distortions owing in part to the restricted bite angle of the nitrate ligand as manifested in the O1—Cd—O2 angle of 52.80 (7)°. A more regular geometry was found in the anhydrous form of the complex (Hazari *et al.*, 2010). The N—H atoms are orientated oppositely going around the macrocyclic ring. The non-coordinating nitrate anion straddles one side of the cation forming two N—H···O hydrogen bonds and an eight-membered {···ONO···HNCdNH} synthon. The formation of N—H···O hydrogen bonds between a third amine-H and an oxygen atom of the coordinated nitrate ligand leads to four-ion aggregates. These are linked into a supramolecular layer in the *bc* plane *via* C—H···O interactions involving the non-coordinating nitrate anion, Fig. 2 and Table 2. The water molecules link layers in the *a* direction forming O—H···O hydrogen bonds with the O5 atom of the non-coordinating nitrate anion and C—H···O interactions, Fig. 3 and Table 2.

### Experimental

The macrocyclic ligand, 3,10-*C*-*meso*-2,5,5,7,9,12,12,14-octamethyl-1,4,8,11-tetraazacyclotetradecane (0.312 g, 1.0 mmol), prepared in accord with the literature procedure (Bembi *et al.*, 1989), was dissolved in methanol (20 ml) in a round bottomed flask. Cadmium(II) nitrate hexahydrate (0.344 g, 1.0 mmol) in methanol (20 ml) was added drop wise to the round bottom flask with continuous stirring The mixture was heated for about 30 min. on a steam bath to ensure the completion of the reaction and was then filtered. After 48 h, the white crystalline product that formed from the filtrate was filtered off, washed with methanol followed by diethylether and dried in a desiccator over silica gel. Yield 85%. *M*.pt: 515–518 K. Anal. Calc. for. C_18_H_41_CdN_6_O_6.5_ C, 38.75; H, 7.41; N, 15.06; Cd, 20.15%. Found: C, 38.85; H, 7.33; N, 15.75; Cd, 20.35%. FT—IR (KBr, cm^-1^) 3200 ν(N—H), 2980 ν(C—H), 1371 ν(CH_3_), 1178 ν(C—C), 520 ν(Cd—N), 1381, 1460, 1275, 730, 820 ν(NO_3_).

### Refinement

The N– and C-bound H-atoms were placed in calculated positions (N—H = 0.88 Å and C—H = 0.95–0.99 Å) and were included in the refinement in the riding model approximation, with *U*_iso_(H) = 1.2-1.5*U*_eq_(N,C). The O—H atom was located from a difference map and refined with O—H = 0.84±0.01 Å, and with *U*_iso_(H)= 1.5*U*_eq_(O).

### Crystal data

**Table:**

[Cd(NO_3_)(C_18_H_40_N_4_)]NO_3_·0.5H_2_O	*F*(000) = 2328
*M**_r_* = 557.98	*D*_x_ = 1.515 Mg m^−^^3^
Monoclinic, *C*2/*c*	Cu *K*α radiation, λ = 1.54184 Å
Hall symbol: -C 2yc	Cell parameters from 4995 reflections
*a* = 18.4312 (4) Å	θ = 3.8–74.2°
*b* = 11.3595 (2) Å	µ = 7.55 mm^−^^1^
*c* = 25.1662 (6) Å	*T* = 100 K
β = 111.782 (3)°	Block, colourless
*V* = 4892.8 (2) Å^3^	0.15 × 0.15 × 0.15 mm
*Z* = 8	

### Data collection

**Table:**

Agilent SuperNova Dual diffractometer with an Atlas detector	4713 independent reflections
Radiation source: SuperNova (Cu) X-ray Source	4467 reflections with *I* > 2σ(*I*)
Mirror monochromator	*R*_int_ = 0.019
Detector resolution: 10.4041 pixels mm^-1^	θ_max_ = 72.5°, θ_min_ = 3.8°
ω scan	*h* = −22→22
Absorption correction: multi-scan (*CrysAlis PRO*; Agilent, 2011)	*k* = −13→10
*T*_min_ = 0.738, *T*_max_ = 1.000	*l* = −26→30
8065 measured reflections	

### Refinement

**Table:**

Refinement on *F*^2^	Primary atom site location: structure-invariant direct methods
Least-squares matrix: full	Secondary atom site location: difference Fourier map
*R*[*F*^2^ > 2σ(*F*^2^)] = 0.029	Hydrogen site location: inferred from neighbouring sites
*wR*(*F*^2^) = 0.078	H atoms treated by a mixture of independent and constrained refinement
*S* = 1.06	*w* = 1/[σ^2^(*F*_o_^2^) + (0.0464*P*)^2^ + 5.776*P*] where *P* = (*F*_o_^2^ + 2*F*_c_^2^)/3
4713 reflections	(Δ/σ)_max_ = 0.003
289 parameters	Δρ_max_ = 0.81 e Å^−^^3^
1 restraint	Δρ_min_ = −0.63 e Å^−^^3^

### Special details

**Table:**

Geometry. All e.s.d.'s (except the e.s.d. in the dihedral angle between two l.s. planes) are estimated using the full covariance matrix. The cell e.s.d.'s are taken into account individually in the estimation of e.s.d.'s in distances, angles and torsion angles; correlations between e.s.d.'s in cell parameters are only used when they are defined by crystal symmetry. An approximate (isotropic) treatment of cell e.s.d.'s is used for estimating e.s.d.'s involving l.s. planes.
Refinement. Refinement of *F*^2^ against ALL reflections. The weighted *R*-factor *wR* and goodness of fit *S* are based on *F*^2^, conventional *R*-factors *R* are based on *F*, with *F* set to zero for negative *F*^2^. The threshold expression of *F*^2^ > σ(*F*^2^) is used only for calculating *R*-factors(gt) *etc*. and is not relevant to the choice of reflections for refinement. *R*-factors based on *F*^2^ are statistically about twice as large as those based on *F*, and *R*- factors based on ALL data will be even larger.

### Fractional atomic coordinates and isotropic or equivalent isotropic displacement parameters (Å^2^)

**Table:**

	*x*	*y*	*z*	*U*_iso_*/*U*_eq_	
Cd	0.197738 (9)	0.571407 (14)	0.583635 (7)	0.01711 (7)	
O1	0.14876 (11)	0.69017 (17)	0.49587 (8)	0.0267 (4)	
O2	0.09653 (11)	0.51918 (19)	0.49425 (8)	0.0303 (4)	
O3	0.05888 (12)	0.6264 (2)	0.41718 (8)	0.0349 (5)	
O4	0.30600 (16)	0.5939 (2)	0.78128 (12)	0.0541 (7)	
O5	0.36324 (14)	0.4804 (2)	0.74149 (9)	0.0411 (5)	
O6	0.42998 (16)	0.5642 (3)	0.82134 (12)	0.0625 (9)	
O1w	0.5000	0.3546 (3)	0.7500	0.0546 (10)	
H1w	0.468 (2)	0.400 (3)	0.757 (2)	0.071 (15)*	
N1	0.31753 (12)	0.59274 (19)	0.57539 (9)	0.0180 (4)	
H1n	0.3114	0.6462	0.5488	0.022*	
N2	0.26080 (13)	0.69852 (18)	0.65796 (9)	0.0204 (4)	
H2n	0.2802	0.6540	0.6886	0.024*	
N3	0.10838 (12)	0.5348 (2)	0.62575 (9)	0.0201 (4)	
H3n	0.0670	0.5039	0.5991	0.024*	
N4	0.24524 (12)	0.39622 (19)	0.63040 (9)	0.0185 (4)	
H4n	0.2805	0.4164	0.6638	0.022*	
N5	0.10019 (11)	0.6129 (2)	0.46812 (9)	0.0201 (4)	
N6	0.36913 (15)	0.5470 (2)	0.78257 (10)	0.0305 (5)	
C1	0.37042 (15)	0.6450 (2)	0.63004 (11)	0.0224 (5)	
H1A	0.4184	0.6743	0.6255	0.027*	
H1B	0.3858	0.5841	0.6603	0.027*	
C2	0.32955 (15)	0.7467 (2)	0.64762 (11)	0.0240 (5)	
H2A	0.3101	0.8032	0.6149	0.029*	
C3	0.38695 (18)	0.8119 (3)	0.69901 (13)	0.0349 (7)	
H3A	0.3599	0.8767	0.7096	0.052*	
H3B	0.4298	0.8434	0.6891	0.052*	
H3C	0.4080	0.7574	0.7313	0.052*	
C4	0.20833 (16)	0.7847 (2)	0.67095 (12)	0.0250 (5)	
H4A	0.2421	0.8293	0.7055	0.030*	
C5	0.17717 (18)	0.8748 (3)	0.62320 (13)	0.0304 (6)	
H5A	0.2209	0.9107	0.6159	0.046*	
H5B	0.1483	0.9359	0.6345	0.046*	
H5C	0.1423	0.8358	0.5884	0.046*	
C6	0.14814 (16)	0.7182 (2)	0.68874 (12)	0.0258 (6)	
H6A	0.1235	0.7779	0.7053	0.031*	
H6B	0.1786	0.6658	0.7206	0.031*	
C7	0.08049 (16)	0.6424 (2)	0.64788 (12)	0.0247 (5)	
C8	0.02921 (17)	0.7092 (3)	0.59449 (13)	0.0312 (6)	
H8A	−0.0124	0.6573	0.5703	0.047*	
H8B	0.0611	0.7354	0.5731	0.047*	
H8C	0.0060	0.7778	0.6058	0.047*	
C9	0.02975 (18)	0.6059 (3)	0.68196 (14)	0.0318 (6)	
H9A	−0.0140	0.5574	0.6577	0.048*	
H9B	0.0095	0.6764	0.6941	0.048*	
H9C	0.0615	0.5606	0.7157	0.048*	
C10	0.14391 (16)	0.4404 (2)	0.66829 (12)	0.0226 (5)	
H10A	0.1034	0.4060	0.6807	0.027*	
H10B	0.1851	0.4748	0.7023	0.027*	
C11	0.17965 (15)	0.3427 (2)	0.64345 (11)	0.0215 (5)	
H11	0.2019	0.2813	0.6736	0.026*	
C12	0.11974 (15)	0.2840 (2)	0.59113 (12)	0.0251 (5)	
H12A	0.1453	0.2224	0.5771	0.038*	
H12B	0.0970	0.3429	0.5611	0.038*	
H12C	0.0783	0.2489	0.6016	0.038*	
C13	0.28665 (14)	0.3164 (2)	0.60372 (11)	0.0203 (5)	
H13	0.2493	0.2937	0.5648	0.024*	
C14	0.31561 (16)	0.2039 (2)	0.63891 (12)	0.0263 (6)	
H14A	0.2713	0.1628	0.6431	0.039*	
H14B	0.3537	0.2245	0.6768	0.039*	
H14C	0.3404	0.1524	0.6193	0.039*	
C15	0.35667 (14)	0.3792 (2)	0.59731 (11)	0.0223 (5)	
H15A	0.3872	0.3186	0.5864	0.027*	
H15B	0.3901	0.4077	0.6358	0.027*	
C16	0.34558 (14)	0.4833 (2)	0.55567 (11)	0.0211 (5)	
C17	0.28420 (16)	0.4545 (2)	0.49697 (11)	0.0243 (5)	
H17A	0.2781	0.5218	0.4713	0.037*	
H17B	0.2342	0.4378	0.5008	0.037*	
H17C	0.3009	0.3854	0.4811	0.037*	
C18	0.42393 (15)	0.5078 (3)	0.54909 (13)	0.0272 (6)	
H18A	0.4177	0.5737	0.5226	0.041*	
H18B	0.4404	0.4375	0.5340	0.041*	
H18C	0.4636	0.5277	0.5865	0.041*	

### Atomic displacement parameters (Å^2^)

**Table:**

	*U*^11^	*U*^22^	*U*^33^	*U*^12^	*U*^13^	*U*^23^
Cd	0.01787 (10)	0.01742 (11)	0.01620 (11)	0.00284 (6)	0.00652 (7)	−0.00031 (6)
O1	0.0250 (9)	0.0259 (10)	0.0247 (10)	0.0008 (8)	0.0042 (8)	0.0014 (8)
O2	0.0318 (10)	0.0334 (11)	0.0253 (10)	−0.0033 (9)	0.0101 (8)	0.0040 (8)
O3	0.0285 (10)	0.0479 (13)	0.0194 (10)	0.0028 (9)	−0.0014 (8)	0.0061 (9)
O4	0.0538 (16)	0.0466 (14)	0.0562 (17)	0.0203 (12)	0.0135 (13)	−0.0026 (13)
O5	0.0483 (13)	0.0455 (14)	0.0239 (11)	−0.0074 (11)	0.0067 (9)	−0.0056 (10)
O6	0.0405 (14)	0.099 (3)	0.0364 (14)	−0.0271 (14)	0.0015 (11)	−0.0104 (14)
O1w	0.063 (2)	0.0305 (18)	0.089 (3)	0.000	0.051 (2)	0.000
N1	0.0179 (10)	0.0188 (10)	0.0172 (10)	0.0034 (8)	0.0064 (8)	0.0007 (8)
N2	0.0268 (11)	0.0164 (10)	0.0186 (10)	0.0009 (9)	0.0092 (8)	−0.0017 (8)
N3	0.0202 (10)	0.0212 (10)	0.0204 (10)	0.0033 (9)	0.0094 (8)	−0.0015 (9)
N4	0.0186 (9)	0.0174 (10)	0.0194 (10)	0.0014 (8)	0.0069 (8)	−0.0012 (8)
N5	0.0128 (9)	0.0288 (12)	0.0185 (10)	0.0050 (9)	0.0055 (8)	0.0030 (9)
N6	0.0420 (14)	0.0243 (12)	0.0213 (12)	−0.0032 (11)	0.0071 (10)	0.0054 (10)
C1	0.0210 (12)	0.0241 (13)	0.0205 (12)	−0.0023 (10)	0.0058 (10)	−0.0018 (10)
C2	0.0281 (13)	0.0222 (13)	0.0225 (12)	−0.0039 (11)	0.0102 (10)	−0.0040 (10)
C3	0.0395 (16)	0.0367 (17)	0.0288 (15)	−0.0147 (13)	0.0129 (13)	−0.0109 (13)
C4	0.0350 (14)	0.0207 (13)	0.0240 (13)	0.0009 (11)	0.0163 (11)	−0.0045 (10)
C5	0.0426 (16)	0.0229 (14)	0.0307 (15)	0.0045 (12)	0.0194 (13)	0.0006 (11)
C6	0.0351 (14)	0.0239 (13)	0.0242 (13)	0.0026 (11)	0.0178 (11)	−0.0036 (11)
C7	0.0283 (13)	0.0236 (13)	0.0270 (13)	0.0059 (11)	0.0160 (11)	−0.0023 (11)
C8	0.0297 (14)	0.0266 (14)	0.0378 (16)	0.0110 (12)	0.0133 (12)	0.0027 (12)
C9	0.0355 (15)	0.0301 (15)	0.0398 (17)	0.0049 (13)	0.0257 (14)	−0.0042 (13)
C10	0.0245 (13)	0.0223 (13)	0.0236 (13)	0.0035 (10)	0.0120 (11)	0.0016 (10)
C11	0.0220 (12)	0.0186 (12)	0.0256 (13)	0.0017 (10)	0.0109 (10)	0.0015 (10)
C12	0.0234 (12)	0.0236 (13)	0.0310 (14)	−0.0008 (10)	0.0132 (11)	−0.0038 (11)
C13	0.0202 (11)	0.0173 (12)	0.0238 (12)	0.0044 (10)	0.0086 (10)	−0.0011 (10)
C14	0.0276 (13)	0.0204 (13)	0.0328 (15)	0.0070 (11)	0.0133 (11)	0.0031 (11)
C15	0.0182 (11)	0.0229 (13)	0.0269 (13)	0.0046 (10)	0.0095 (10)	0.0012 (11)
C16	0.0193 (11)	0.0233 (13)	0.0221 (12)	0.0036 (10)	0.0095 (10)	−0.0008 (10)
C17	0.0262 (13)	0.0264 (13)	0.0214 (13)	0.0030 (11)	0.0101 (11)	−0.0020 (11)
C18	0.0218 (12)	0.0296 (14)	0.0344 (15)	0.0043 (11)	0.0153 (11)	0.0024 (12)

### Geometric parameters (Å, º)

**Table:**

Cd—O1	2.4562 (19)	C5—H5C	0.9800
Cd—O2	2.404 (2)	C6—C7	1.550 (4)
Cd—N1	2.306 (2)	C6—H6A	0.9900
Cd—N2	2.307 (2)	C6—H6B	0.9900
Cd—N3	2.303 (2)	C7—C8	1.527 (4)
Cd—N4	2.312 (2)	C7—C9	1.542 (4)
O1—N5	1.263 (3)	C8—H8A	0.9800
O2—N5	1.266 (3)	C8—H8B	0.9800
O3—N5	1.234 (3)	C8—H8C	0.9800
O4—N6	1.269 (4)	C9—H9A	0.9800
O5—N6	1.253 (3)	C9—H9B	0.9800
O6—N6	1.198 (4)	C9—H9C	0.9800
O1w—H1w	0.850 (10)	C10—C11	1.537 (3)
N1—C1	1.483 (3)	C10—H10A	0.9900
N1—C16	1.500 (3)	C10—H10B	0.9900
N1—H1n	0.8800	C11—C12	1.523 (4)
N2—C2	1.489 (3)	C11—H11	1.0000
N2—C4	1.495 (3)	C12—H12A	0.9800
N2—H2n	0.8800	C12—H12B	0.9800
N3—C10	1.485 (3)	C12—H12C	0.9800
N3—C7	1.511 (3)	C13—C14	1.534 (3)
N3—H3n	0.8800	C13—C15	1.535 (3)
N4—C11	1.494 (3)	C13—H13	1.0000
N4—C13	1.496 (3)	C14—H14A	0.9800
N4—H4n	0.8800	C14—H14B	0.9800
C1—C2	1.531 (4)	C14—H14C	0.9800
C1—H1A	0.9900	C15—C16	1.542 (4)
C1—H1B	0.9900	C15—H15A	0.9900
C2—C3	1.525 (4)	C15—H15B	0.9900
C2—H2A	1.0000	C16—C17	1.526 (4)
C3—H3A	0.9800	C16—C18	1.539 (3)
C3—H3B	0.9800	C17—H17A	0.9800
C3—H3C	0.9800	C17—H17B	0.9800
C4—C5	1.519 (4)	C17—H17C	0.9800
C4—C6	1.540 (4)	C18—H18A	0.9800
C4—H4A	1.0000	C18—H18B	0.9800
C5—H5A	0.9800	C18—H18C	0.9800
C5—H5B	0.9800		
			
N3—Cd—N1	158.83 (8)	C4—C6—H6A	106.2
N3—Cd—N2	88.35 (8)	C7—C6—H6A	106.2
N1—Cd—N2	78.23 (7)	C4—C6—H6B	106.2
N3—Cd—N4	79.08 (7)	C7—C6—H6B	106.2
N1—Cd—N4	86.67 (7)	H6A—C6—H6B	106.4
N2—Cd—N4	98.30 (7)	N3—C7—C8	105.2 (2)
N3—Cd—O2	86.96 (7)	N3—C7—C9	110.3 (2)
N1—Cd—O2	112.22 (7)	C8—C7—C9	108.6 (2)
N2—Cd—O2	153.62 (7)	N3—C7—C6	113.2 (2)
N4—Cd—O2	106.24 (7)	C8—C7—C6	113.0 (2)
N3—Cd—O1	115.22 (7)	C9—C7—C6	106.6 (2)
N1—Cd—O1	84.61 (7)	C7—C8—H8A	109.5
N2—Cd—O1	106.96 (7)	C7—C8—H8B	109.5
N4—Cd—O1	150.90 (7)	H8A—C8—H8B	109.5
O2—Cd—O1	52.80 (7)	C7—C8—H8C	109.5
N5—O1—Cd	93.68 (14)	H8A—C8—H8C	109.5
N5—O2—Cd	96.09 (15)	H8B—C8—H8C	109.5
C1—N1—C16	116.76 (19)	C7—C9—H9A	109.5
C1—N1—Cd	106.29 (15)	C7—C9—H9B	109.5
C16—N1—Cd	113.69 (15)	H9A—C9—H9B	109.5
C1—N1—H1n	106.5	C7—C9—H9C	109.5
C16—N1—H1n	106.5	H9A—C9—H9C	109.5
Cd—N1—H1n	106.5	H9B—C9—H9C	109.5
C2—N2—C4	117.3 (2)	N3—C10—C11	111.7 (2)
C2—N2—Cd	107.09 (15)	N3—C10—H10A	109.3
C4—N2—Cd	114.53 (16)	C11—C10—H10A	109.3
C2—N2—H2n	105.6	N3—C10—H10B	109.3
C4—N2—H2n	105.6	C11—C10—H10B	109.3
Cd—N2—H2n	105.6	H10A—C10—H10B	107.9
C10—N3—C7	116.0 (2)	N4—C11—C12	112.0 (2)
C10—N3—Cd	105.24 (15)	N4—C11—C10	107.4 (2)
C7—N3—Cd	115.00 (16)	C12—C11—C10	112.6 (2)
C10—N3—H3n	106.7	N4—C11—H11	108.2
C7—N3—H3n	106.7	C12—C11—H11	108.2
Cd—N3—H3n	106.7	C10—C11—H11	108.2
C11—N4—C13	116.2 (2)	C11—C12—H12A	109.5
C11—N4—Cd	106.12 (14)	C11—C12—H12B	109.5
C13—N4—Cd	116.95 (15)	H12A—C12—H12B	109.5
C11—N4—H4n	105.5	C11—C12—H12C	109.5
C13—N4—H4n	105.5	H12A—C12—H12C	109.5
Cd—N4—H4n	105.5	H12B—C12—H12C	109.5
O3—N5—O1	121.7 (2)	N4—C13—C14	111.9 (2)
O3—N5—O2	120.8 (2)	N4—C13—C15	110.8 (2)
O1—N5—O2	117.4 (2)	C14—C13—C15	108.8 (2)
O6—N6—O5	122.6 (3)	N4—C13—H13	108.4
O6—N6—O4	121.7 (3)	C14—C13—H13	108.4
O5—N6—O4	115.8 (3)	C15—C13—H13	108.4
N1—C1—C2	110.2 (2)	C13—C14—H14A	109.5
N1—C1—H1A	109.6	C13—C14—H14B	109.5
C2—C1—H1A	109.6	H14A—C14—H14B	109.5
N1—C1—H1B	109.6	C13—C14—H14C	109.5
C2—C1—H1B	109.6	H14A—C14—H14C	109.5
H1A—C1—H1B	108.1	H14B—C14—H14C	109.5
N2—C2—C3	113.4 (2)	C13—C15—C16	121.5 (2)
N2—C2—C1	108.3 (2)	C13—C15—H15A	106.9
C3—C2—C1	110.5 (2)	C16—C15—H15A	106.9
N2—C2—H2A	108.1	C13—C15—H15B	106.9
C3—C2—H2A	108.1	C16—C15—H15B	106.9
C1—C2—H2A	108.1	H15A—C15—H15B	106.7
C2—C3—H3A	109.5	N1—C16—C17	106.0 (2)
C2—C3—H3B	109.5	N1—C16—C18	109.8 (2)
H3A—C3—H3B	109.5	C17—C16—C18	108.8 (2)
C2—C3—H3C	109.5	N1—C16—C15	112.7 (2)
H3A—C3—H3C	109.5	C17—C16—C15	110.9 (2)
H3B—C3—H3C	109.5	C18—C16—C15	108.6 (2)
N2—C4—C5	110.7 (2)	C16—C17—H17A	109.5
N2—C4—C6	109.7 (2)	C16—C17—H17B	109.5
C5—C4—C6	117.3 (2)	H17A—C17—H17B	109.5
N2—C4—H4A	106.2	C16—C17—H17C	109.5
C5—C4—H4A	106.2	H17A—C17—H17C	109.5
C6—C4—H4A	106.2	H17B—C17—H17C	109.5
C4—C5—H5A	109.5	C16—C18—H18A	109.5
C4—C5—H5B	109.5	C16—C18—H18B	109.5
H5A—C5—H5B	109.5	H18A—C18—H18B	109.5
C4—C5—H5C	109.5	C16—C18—H18C	109.5
H5A—C5—H5C	109.5	H18A—C18—H18C	109.5
H5B—C5—H5C	109.5	H18B—C18—H18C	109.5
C4—C6—C7	124.7 (2)		
			
N3—Cd—O1—N5	−65.15 (15)	Cd—O1—N5—O3	−177.3 (2)
N1—Cd—O1—N5	122.59 (14)	Cd—O1—N5—O2	1.2 (2)
N2—Cd—O1—N5	−161.48 (13)	Cd—O2—N5—O3	177.29 (19)
N4—Cd—O1—N5	49.4 (2)	Cd—O2—N5—O1	−1.2 (2)
O2—Cd—O1—N5	−0.68 (12)	C16—N1—C1—C2	−173.3 (2)
N3—Cd—O2—N5	125.85 (15)	Cd—N1—C1—C2	−45.3 (2)
N1—Cd—O2—N5	−63.37 (16)	C4—N2—C2—C3	61.9 (3)
N2—Cd—O2—N5	45.8 (2)	Cd—N2—C2—C3	−167.8 (2)
N4—Cd—O2—N5	−156.45 (14)	C4—N2—C2—C1	−175.0 (2)
O1—Cd—O2—N5	0.68 (12)	Cd—N2—C2—C1	−44.7 (2)
N3—Cd—N1—C1	−36.2 (3)	N1—C1—C2—N2	63.1 (3)
N2—Cd—N1—C1	15.51 (15)	N1—C1—C2—C3	−172.0 (2)
N4—Cd—N1—C1	−83.68 (16)	C2—N2—C4—C5	57.1 (3)
O2—Cd—N1—C1	170.12 (15)	Cd—N2—C4—C5	−69.7 (2)
O1—Cd—N1—C1	124.11 (16)	C2—N2—C4—C6	−171.9 (2)
N3—Cd—N1—C16	93.6 (2)	Cd—N2—C4—C6	61.3 (2)
N2—Cd—N1—C16	145.33 (17)	N2—C4—C6—C7	−69.3 (3)
N4—Cd—N1—C16	46.13 (16)	C5—C4—C6—C7	58.0 (4)
O2—Cd—N1—C16	−60.06 (17)	C10—N3—C7—C8	−166.0 (2)
O1—Cd—N1—C16	−106.07 (16)	Cd—N3—C7—C8	70.6 (2)
N3—Cd—N2—C2	179.50 (17)	C10—N3—C7—C9	−49.2 (3)
N1—Cd—N2—C2	15.96 (16)	Cd—N3—C7—C9	−172.55 (18)
N4—Cd—N2—C2	100.78 (16)	C10—N3—C7—C6	70.1 (3)
O2—Cd—N2—C2	−100.7 (2)	Cd—N3—C7—C6	−53.3 (3)
O1—Cd—N2—C2	−64.60 (17)	C4—C6—C7—N3	65.4 (3)
N3—Cd—N2—C4	−48.61 (17)	C4—C6—C7—C8	−54.0 (3)
N1—Cd—N2—C4	147.85 (18)	C4—C6—C7—C9	−173.2 (2)
N4—Cd—N2—C4	−127.33 (17)	C7—N3—C10—C11	−172.6 (2)
O2—Cd—N2—C4	31.1 (3)	Cd—N3—C10—C11	−44.3 (2)
O1—Cd—N2—C4	67.29 (17)	C13—N4—C11—C12	−53.8 (3)
N1—Cd—N3—C10	−35.0 (3)	Cd—N4—C11—C12	78.1 (2)
N2—Cd—N3—C10	−85.27 (16)	C13—N4—C11—C10	−178.0 (2)
N4—Cd—N3—C10	13.51 (16)	Cd—N4—C11—C10	−46.1 (2)
O2—Cd—N3—C10	120.70 (16)	N3—C10—C11—N4	64.2 (3)
O1—Cd—N3—C10	166.73 (15)	N3—C10—C11—C12	−59.6 (3)
N1—Cd—N3—C7	93.9 (3)	C11—N4—C13—C14	−53.7 (3)
N2—Cd—N3—C7	43.65 (17)	Cd—N4—C13—C14	179.69 (16)
N4—Cd—N3—C7	142.43 (18)	C11—N4—C13—C15	−175.2 (2)
O2—Cd—N3—C7	−110.38 (17)	Cd—N4—C13—C15	58.1 (2)
O1—Cd—N3—C7	−64.35 (18)	N4—C13—C15—C16	−67.4 (3)
N3—Cd—N4—C11	18.04 (15)	C14—C13—C15—C16	169.3 (2)
N1—Cd—N4—C11	−177.69 (16)	C1—N1—C16—C17	−175.4 (2)
N2—Cd—N4—C11	104.73 (16)	Cd—N1—C16—C17	60.3 (2)
O2—Cd—N4—C11	−65.50 (16)	C1—N1—C16—C18	−58.0 (3)
O1—Cd—N4—C11	−105.02 (18)	Cd—N1—C16—C18	177.63 (16)
N3—Cd—N4—C13	149.51 (18)	C1—N1—C16—C15	63.1 (3)
N1—Cd—N4—C13	−46.22 (17)	Cd—N1—C16—C15	−61.2 (2)
N2—Cd—N4—C13	−123.81 (17)	C13—C15—C16—N1	70.9 (3)
O2—Cd—N4—C13	65.97 (18)	C13—C15—C16—C17	−47.8 (3)
O1—Cd—N4—C13	26.4 (3)	C13—C15—C16—C18	−167.3 (2)

### Hydrogen-bond geometry (Å, º)

**Table:**

*D*—H···*A*	*D*—H	H···*A*	*D*···*A*	*D*—H···*A*
O1*w*—H1*w*···O5	0.85 (1)	2.04 (2)	2.836 (3)	156 (5)
N1—H1*n*···O1^i^	0.88	2.42	3.242 (3)	155
N2—H2*n*···O4	0.88	2.30	3.133 (4)	157
N4—H4*n*···O5	0.88	2.11	2.991 (3)	175
C5—H5*B*···O6^ii^	0.98	2.58	3.539 (5)	168
C11—H11···O4^iii^	1.00	2.44	3.358 (3)	152
C9—H9*B*···O1*w*^iv^	0.98	2.51	3.451 (4)	162

Symmetry codes: (i) −*x*+1/2, −*y*+3/2, −*z*+1; (ii) −*x*+1/2, *y*+1/2, −*z*+3/2; (iii) −*x*+1/2, *y*−1/2, −*z*+3/2; (iv) *x*−1/2, *y*+1/2, *z*.
